# Gene therapy targeting inflammatory pericytes corrects angiopathy during diabetic wound healing

**DOI:** 10.3389/fimmu.2022.960925

**Published:** 2022-08-03

**Authors:** Wenxv Jin, Xiong Chen, Lingguo Kong, Chongqing Huang

**Affiliations:** ^1^ Department of Vascular Surgery, the First Affiliated Hospital of Wenzhou Medical University, Wenzhou, China; ^2^ Department of Endocrinology, the First Affiliated Hospital of Wenzhou Medical University, Wenzhou, China

**Keywords:** diabetic wound healing, pericytes, diabetes, OXR1, apoptosis, oxidative stress

## Abstract

Wound healing is impaired in the diabetic status, largely attributable to diabetes-associated angiopathy. Pericytes play critical roles in the stabilization of the formed vessels. The loss and dysfunction of pericytes have been reported in inflammation during diabetes and associated with the pathology of diabetic angiopathy. However, a practical approach that targets inflammatory pericytes to improve diabetic wound healing is lacking. In the current study, we showed that the inflammatory pericytes from wound skin of diabetic patients were impaired in growth potential and underwent oxidative stress and apoptosis. Expression of antioxidant gene oxidation resistance protein 1 (OXR1) specifically in pericytes through an adenovirus carrying OXR1 under a pericyte-specific neuron glia antigen-2 (NG2) promoter (AV-NG2p-OXR1) relieved the oxidative stress, reduced the apoptosis, and recovered the growth potential in diabetic pericytes. Moreover, expression of OXR1 in diabetic pericytes retrieved their potential of both suppressing the migration of co-cultured HUVECs and inducing cell aggregates at the branching points, indicating a functional recovery. *In vivo* gene therapy using this AV-NG2p-OXR1 to DB/DB mice, the mouse model for type 2 diabetes, significantly improved wound healing, likely through enhancing blood flow at the wound rather than increasing vessel density. Together, our data suggest that gene therapy targeting inflammatory pericytes may improve diabetes-associated impaired wound healing.

## Introduction

Diabetic angiopathy refers to the dysfunction and impairment of the arteries throughout the body, caused by diabetic status. Diabetic angiopathy affects both microvascular system in feet, fingers, toes, eyes, and kidneys, and macrovascular system in limbs (1).

Pericytes are important perivascular or mural cells that contribute to the proper formation of the microvasculature system. The interaction between pericyte and endothelial cell (EC) plays a pivotal role in the formation and homeostasis of a functional vasculature (2). Pericytes express some specific surface markers, like proteoglycan neuron glial antigen-2 (NG2), platelet-derived growth factor (PDGF) receptor β (PDGFRβ), CD146, or alpha smooth muscle actin (α-SMA) (2). In diabetes, especially in complications associated with vascular disorders, the interaction between pericytes and ECs is disrupted (3). In fact, loss of pericytes has been used as an early hallmark of diabetic retinopathy and nephropathy (4). It is nowadays understood that dysfunctional pericytes promote the progression of diabetic angiopathy through compromised vessel stability, abnormal and inadequate neovascularization, vasoconstriction, and basement membrane thickening (4).

Wound healing is impaired in the diabetic status, largely attributable to the diabetes-associated angiopathy (5). Since pericytes play critical roles in the stabilization of the pre-existing and newly formed vessels, the loss and dysfunction of pericytes in diabetes actively contribute to the impaired wound healing (6). However, so far, a practical approach to improve diabetic wound healing through pericytes is lacking.

Oxidative stress occurs in many cell types under a diabetic status. Oxidation resistance protein 1 (OXR1) is an important protein regulating the sensitivity of cells to oxidative stress and has been shown to have protective effects against oxidation in neural cells (7). In the current study, we showed that the pericytes from wound skin of diabetic patients were impaired in growth potential and underwent oxidative stress and apoptosis. Expression of OXR1 specifically in pericytes through an adenovirus carrying OXR1 under a pericyte-specific NG2 promoter (AV-NG2p-OXR1) relieved the oxidative stress, reduced the apoptosis and recovered the growth potential in diabetic pericytes, and retrieved their potential of supporting vascular stability. *In vivo* gene therapy using this AV-NG2p-OXR1 to DB/DB mice, the mouse model for type 2 diabetes, significantly improved wound healing, likely through enhancing blood flow at the wound rather than increasing vessel density.

## Materials and methods

### Ethical approval of research protocols

This study has been approved by the Research Committee and Institutional Animal Care and Use Committee at the Wenzhou Medical University. Patient specimens were obtained from the inpatients at the First Affiliated Hospital of Wenzhou Medical University with prior signed agreement from the patients.

### Animals

Diabetes automatically occurred in DB/DB mice (SLAC Laboratory Animal, Shanghai, China). Fasting blood sugar was elevated as early as 4 weeks of age and reached the diagnosed level for diabetes (>350 mg/dl) at 8 weeks of age when the mice received wound formation with/without injection of adenovirus. Male and female mice were evenly distributed in each group. For wound generation, mice underwent a surgery to create a wound of approximately 4 mm in diameter on the dorsal side of the right hindlimb using a biopsy punch. Afterwards, the mice received an orthotopic injection of 150 µl of adenoviral virus of 10^12^ genome copy particles (GCP)/ml or 150 µl of saline as a control. Blood glucose was measured after 4-h fasting. Vessel density was assessed by the percentage of the positive immunostaining area for CD31. A laser Doppler perfusion imaging system (LDPI, Moor Instruments, Devon, UK) was used for measurement of blood flow at hindlimbs. Data were presented as a ratio of the wound right side versus non-wound left side.

### Adenovirus

A backbone plasmid (Ng2 promoter-CRE-IRES-RFP) was purchased from Applied Biological Materials Inc. (000844A, Richmond, BC V6V 2J5, Canada) and then modified. The OXR1 coding sequence was cloned from human fibroblast cDNA. A scramble sequence (SCR) was used as a control for the OXR1 transgene. Transfection was performed with Lipofectamine 3000 reagent (Invitrogen, CA, Carlsbad, USA).

### Flow cytometry

The skin tissue obtained from diabetic patients or non-diabetic controls was digested with digesting media containing 0.25% trypsin (Invitrogen) and 10 mg/ml DNase (Invitrogen) for 30–35 min to obtain a single-cell fraction for fluorescence-activated cell sorting (FACS). Pericytes were isolated by FACS based on double-positive immunofluorescence for CD146 (with an FTIC-conjugated anti-CD146 antibody, Becton-Dickinson Biosciences, San Jose, CA, USA) and PDGFRβ (with a cy5-conjugated anti-PDGFRβ antibody). Pericytes from virally infused mice were isolated based on BFP. The flow cytometry data were analyzed and presented by Flowjo (Flowjo LLC, Ashland, OR, USA). Apoptosis was analyzed with an Annexin V-apoptosis analysis kit (Invitrogen).

### Quantitative real-time PCR

RNA was extracted with an RNeasy kit (Qiagen, Beijing, China) to prepare cDNA for real-time quantitative PCR (RT-qPCR) using Qiagen pre-designed primers. A 2^−ΔΔCt^ method was applied for quantification, and β-actin was used as a housekeeping gene for normalization of the expression values for the examined genes.

### Histology and immunostaining

The pancreas and the wound skin were dissected out and fixed in 10% formalin (Sigma-Aldrich, St. Louis, MO, USA) for 5 and 3 h, respectively. After incubation in 30% sucrose for 48 h, the samples were frozen, embedded, and cut into 5-µm slides. Immunofluorescent staining for insulin and CD31 was performed with a guinea pig polyclonal antibody against insulin (Ab7842, Abcam) and a rat-anti-mouse CD31 antibody (Becton-Dickinson Biosciences), respectively. For immunocytochemistry, the blue fluorescence was detected by direct fluorescence from the adenovirus infection.

### Culture, co-culture, cell growth, and migration assay

NOS activity was measured using a nitric oxide synthase assay (Abcam, Los Angeles, CA, USA). Pericytes and HUVECs were co-cultured. Tube formation was quantified by relative number. Branching points were quantified by relative area. Cell aggregates were quantified by relative area containing cell aggregates at branch. Cell growth was determined by a CCK-8 assay (Millipore, Bedford, MA, USA). Cell migration assay was done in an upper chamber (Millipore) that had been seeded with HUVECs and pericytes in serum-free DMEM media, while the lower chamber was filled with DMEM with 7.5% FBS. The migrated cells from the upper chamber to the lower surface were fixed with methanol, and stained with 0.1% crystal violet (Sigma-Aldrich) for quantification after 36-h culture.

### Statistical analysis

Statistical analysis was performed using one-way analysis of variance (ANOVA) test (GraphPad Software, version 9, Inc. La Jolla, CA, USA). Significance (*p* < 0.05) was presented as * and no significance (*p* > 0.05) was presented as “NS”. The number in this experimental group was determined by Power test, and *N* = 5 was selected.

## Results

### Pericytes from wound skin of diabetic patients are impaired

**Figure 1:**
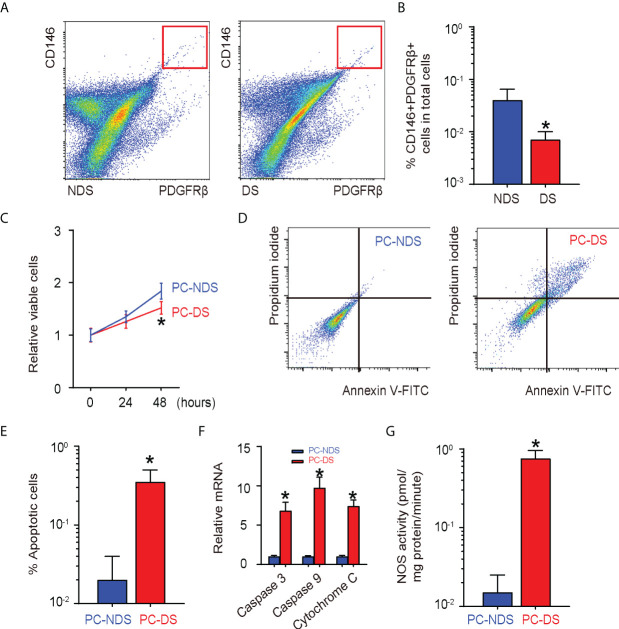
Pericytes from wound skin of diabetic patients are impaired **(A, B)** Wound tissues obtained from non-diabetic wound skin (NDS) and diabetic wound skin (DS) were digested into single cells that underwent fluorescence-activated cell sorting (FACS) for CD146 and platelet-derived growth factor β (PDGFRβ) to isolate CD146+PDGFRβ+ pericytes, shown by representative flowcharts **(A)** and by quantification **(B)**. **(C)** CCK-8 assay for pericytes isolated from NDS (PC-NDS) and NS (PC-NS). **(D,E)** An annexin V apoptosis assay by representative flowcharts **(D)** and by quantification **(E)**. **(F)** RT-qPCR for apoptosis-associated protein Caspase 3, Caspase 9, and Cytochrome 9. **(G)** NOS activity. *p < 0.05. N = 5.

The loss and dysfunction of pericytes cause vascular instability in diabetes and may contribute to impaired wound healing. Here, we aimed to generate a practical approach that targets pericytes to improve diabetic wound healing. First, wound tissues obtained from non-diabetic wound skin (NDS) and diabetic wound skin (DS) were digested into single cells that underwent fluorescence-activated cell sorting (FACS) for CD146 and PDGFRβ to isolate CD146+PDGFRβ+ pericytes (**Figure 1A**). We found that the number of the CD146+PDGFRβ+ pericytes was significantly decreased in DS, compared to NDS (**Figure 1B**). Moreover, the growth potential of pericytes in DS was compromised compared to that in NDS (**Figure 1C**), likely resulting from increased apoptosis (**Figures 1D–F**) and increased NOS activity (**Figure 1G**). Thus, pericytes from wound skin of diabetic patients are impaired in growth potential and undergo oxidative stress and apoptosis.

### OXR1 relieves the oxidative stress, reduces the apoptosis, and recovers the growth potential in diabetic pericytes

**Figure 2:**
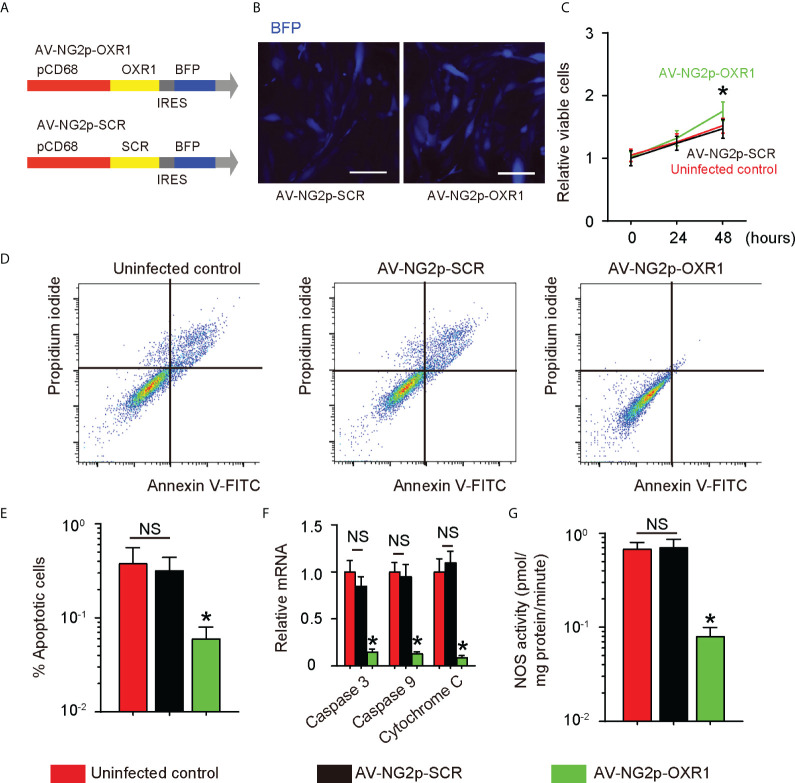
OXR1 relieves the oxidative stress, reduces the apoptosis, and recovers the growth potential in diabetic pericytes. **(A)** Schematic of an adenovirus carrying OXR1 under a pericyte-specific neuron glia antigen-2 (NG2) promoter (AV-NG2p-OXR1) and a control adenovirus carrying a scramble sequence (SCR) under the NG2 promoter (AV-NG2p-SCR). Both viruses also carried a blue fluorescent protein (BFP) as a reporter connected with the transgene with an IRES to be controlled together under the NG2 promoter. **(B)** Pericytes from DS (PC-DS) transduced by both viruses in culture. **(C)** CCK-8 assay for PC-NS transduced with either virus. **(D, E)** An annexin V apoptosis assay by representative flowcharts **(D)** and by quantification **(E)**. **(F)** RT-qPCR for apoptosis-associated protein Caspase 3, Caspase 9, and Cytochrome 9. **(G)** NOS activity. *p < 0.05. NS, no significance. *N* = 5. Scale bars are 10 µm.

To assess the effects of relieving oxidative stress on pericytes, we prepared an adenovirus carrying OXR1 under a pericyte-specific neuron glia antigen-2 (NG2) promoter (AV-NG2p-OXR1) and a control adenovirus carrying a scramble sequence (SCR) under the NG2 promoter (AV-NG2p-SCR). Both viruses also carried a blue fluorescent protein (BFP) as a reporter connected with the transgene with an IRES to be controlled together under the NG2 promoter (**Figure 2A**). Pericytes from DS (PC-DS) were successfully transduced by both viruses (**Figure 2B**). We found that transduction with AV-NG2p-OXR1 significantly increases the growth of PC-DS, compared to either uninfected PC-DS or PC-DS transduced with AV-NG2p-SCR (**Figure 2C**). Moreover, transduction with AV-NG2p-OXR1 significantly reduced the apoptosis of PC-DS cells, by representative flowcharts for an Annexin V apoptosis assay (**Figure 2D**) and its quantification (**Figure 2E**), and by mRNA levels for apoptosis-associated protein Caspase 3, Caspase 9, and Cytochrome 9 (**Figure 2F**). Furthermore, the NOS activity in PC-DS was also significantly reduced by transduction with AV-NG2p-OXR1 (**Figure 2G**). Thus, expression of OXR1 relieves the oxidative stress, reduces the apoptosis and recovers the growth potential in diabetic pericytes.

### OXR1 expression in diabetic pericytes increases aggregates and reduces branching in endothelial cells

**Figure 3:**
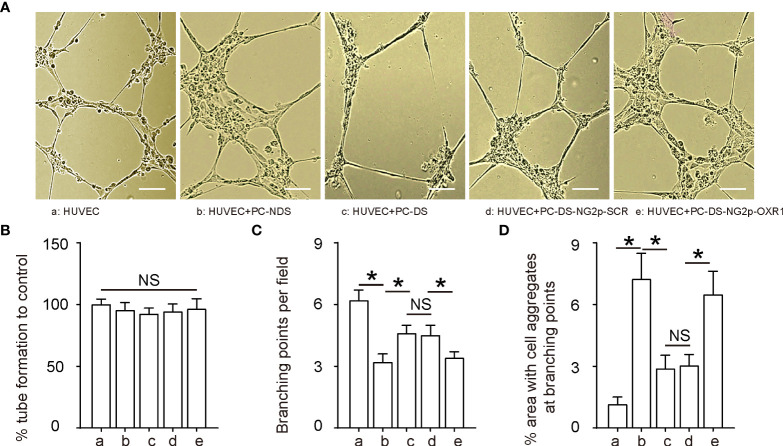
OXR1 expression in diabetic pericytes increases aggregates and reduces branching in endothelial cells To assess the effects of relieving oxidative stress in pericytes on angiogenesis, we used pericytes from NDS (PC-NDS), uninfected PC-DS, and PC-DS transduced with either AV-NG2p-SCR or AV-NG2p-OXR1 to co-culture with HUVECs in a tube formation assay. HUVECs alone in culture were also added as a control. **(A)** Representative images. **(B)** Percent tube formation. **(C)** Branching points. **(D)** Percent area with cell aggregates at branching points. *p < 0.05. NS, no significance. *N* = 5. Scale bars are 50 µm.

To assess the effects of relieving oxidative stress in pericytes on angiogenesis, we used pericytes from NDS (PC-NDS), uninfected PC-DS, and PC-DS transduced with either AV-NG2p-SCR or AV-NG2p-OXR1 to co-culture with HUVECs in a tube formation assay. HUVECs alone in culture were also added as a control. We did not detect the difference in the levels of tube formation in all these conditions (**Figures 3A, B**). However, the branching points were significantly reduced in HUVECs co-cultured with PC-NDS compared to HUVECs alone (**Figures 3A, C**). The number of branching points correlates with the morphological transformation of HUVECs to tube-like structures and their subsequent elongation to generate the vessel-like networks. The branching points in HUVECs co-cultured with PC-DS were significantly increased compared to HUVECs co-cultured with PC-NDS, while this difference was abolished when the co-cultured PC-DS were replaced with PC-DS transduced with AV-NG2p-OXR1 (**Figures 3A, C**). In addition, the area with cell aggregates were significantly increased in HUVECs co-cultured with PC-NDS compared to HUVECs alone (**Figures 3A, D**). The cell aggregates are critical for generation of mature and stable tubular structures. The area with cell aggregates in HUVECs co-cultured with PC-DS was significantly decreased compared to HUVECs co-cultured with PC-NDS, while this difference was abolished when the co-cultured PC-DS was replaced with PC-DS transduced with AV-NG2p-OXR1 (**Figures 3A, D**). Together, these data suggest that OXR1 expression in diabetic pericytes increases aggregates and reduces branching in ECs, restoring the potential of stabilizing vascular structures in diabetic pericytes.

### OXR1 expression in diabetic pericytes decreases the migratory potential of endothelial cells

**Figure 4:**
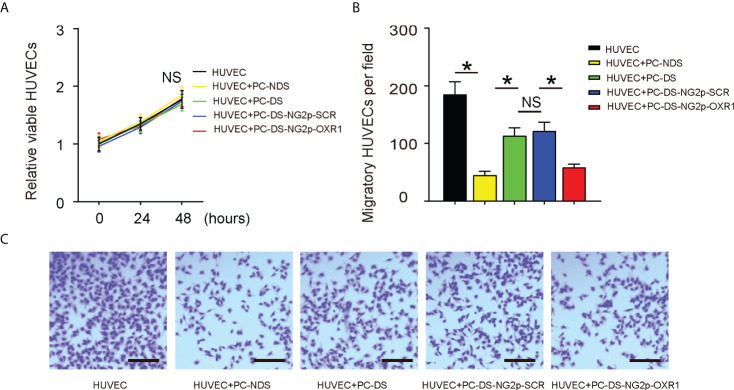
OXR1 expression in diabetic pericytes decreases the migratory potential of endothelial cells. The HUVECs were alone or co-cultured with NDS (PC-NDS), uninfected PC-DS, and PC-DS transduced with either AV-NG2p-SCR or AV-NG2p-OXR1. **(A)** CCK-8 assay. **(B, C)** A cell migration assay, shown by representative images **(B)** and by quantification **(C)**. *p < 0.05. NS, no significance. *N* = 5. Scale bars are 100 µm.

The growth of HUVECs in these five conditions [HUVECs alone, or co-cultured with pericytes from NDS (PC-NDS), with uninfected PC-DS, or with PC-DS transduced with either AV-NG2p-SCR or AV-NG2p-OXR1] was also analyzed, showing no difference among all groups (**Figure 4A**). In a cell migration assay, HUVECs exhibited decreased migratory potential when they were co-cultured with PC-NDS compared to HUVECs alone (**Figures 4B, C**). The migrated cells in HUVECs co-cultured with PC-DS were significantly increased compared to HUVECs co-cultured with PC-NDS, while this difference was attenuated when the co-cultured PC-DS were replaced with PC-DS transduced with AV-NG2p-OXR1 (**Figures 4B, C**). Thus, these data suggest that OXR1 expression in diabetic pericytes decreases the migratory potential of ECs, likely contributing to their regulation of vascular stability and angiogenesis.

### Gene therapy with AV-NG2p-OXR1 does not alter diabetic status in DB/DB mice

**Figure 5:**
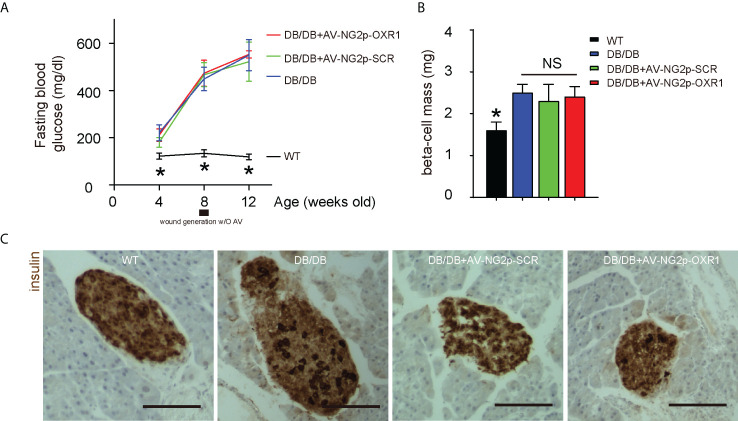
Gene therapy with AV-NG2p-OXR1 does not alter diabetic status in DB/DB mice. DB/DB mice received injection of control saline or control AV-NG2p-SCR or AV-NG2p-OXR1 at the wound site at 8 weeks old when the wound was generated. Another group of wild-type mice was used as an additional control. **(A)** Fasting blood glucose. **(B)** Beta cell mass at analysis (12 weeks old, or 4 weeks after wound generation with/without saline/virus injections). **(C)** Representative immunostaining for insulin. *p < 0.05. NS, no significance. *N* = 5. Scale bars are 100 µm.

Finally, we examined the effects of relieving oxidative stress in pericytes on diabetic wound healing *in vivo*. DB/DB is a mouse model for type 2 diabetes, in which mice develop high fasting blood glucose as early as 8 weeks old in both genders (**Figure 5A**). DB/DB mice received injection of control saline or control AV-NG2p-SCR or AV-NG2p-OXR1 at the wound site at 8 weeks old when the wound was generated. Another group of wild-type mice was used as an additional control. Gene therapy with AV-NG2p-OXR1 did not alter the levels of fasting blood glucose in DB/DB mice (**Figure 5A**) and did not alter beta cell mass at analysis (12 weeks old, or 4 weeks after wound generation with/without saline/virus injections) (**Figures 5B, C**). Thus, gene therapy with AV-NG2p-OXR1 does not alter diabetic status in DB/DB mice.

### Gene therapy with AV-NG2p-OXR1 improves diabetic wound healing, likely through enhancing blood flow at the wound rather than increasing vessel density

**Figure 6:**
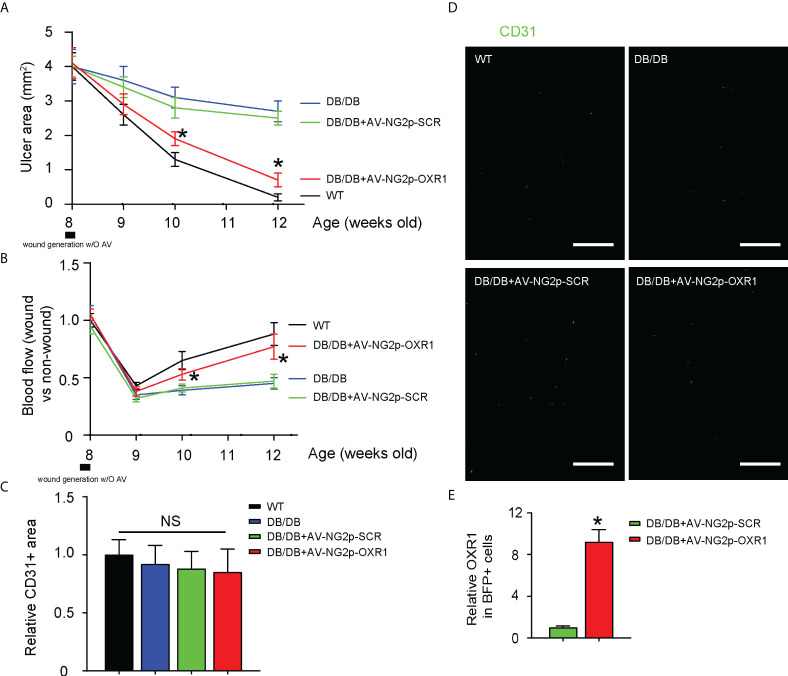
Gene therapy with AV-NG2p-OXR1 improves diabetic wound healing, likely through enhancing blood flow at the wound rather than increasing vessel density. **(A)** Changes in ulcer area with time. **(B)** changes in blood flow with time. **(C, D)** Vessel density at analysis (12 weeks old, or 4 weeks after wound generation with/without saline/virus injections), shown by quantification **(C)** and by representative fluorescent images **(D)**. **(E)** RT-qPCR for OXR1 in BFP+ cells isolated from the wound. *p < 0.05. NS, non-significant. *N* = 5. Scale bars are 100 µm.

Next, we examined the effects of this gene therapy targeting pericytes on wound healing in mice. We found that the wound was completely cured 4 weeks after ulcer induction in wild-type mice and was hardly recovered in DB/DB mice treated with either saline or AV-NG2p-SCR (**Figure 6A**). However, a significant improvement in the recovery was detected in DB/DB mice treated with AV-NG2p-OXR1 (**Figure 6A**). Interestingly, this improvement in diabetic wound healing by AV-NG2p-OXR1 was likely through enhancing blood flow at the wound (**Figure 6B**) rather than increasing vessel density (**Figures 6C, D**). BFP+ cells were isolated from the wound and confirmed the increases in OXR1 levels by AV-NG2p-OXR1 (**Figure 6E**).

## Discussion

A proper wound healing requires coordination of many biological processes including inflammatory reaction and removal of dead tissue/cells, cell differentiation and proliferation, and angiogenesis. Although proliferation, migration, and structuring of ECs are the most important parts for a proper angiogenesis, it is acknowledged that pericytes play a non-redundant role in the stability of the newly formed vascular structures.

In this study, we showed impaired growth potential and augmented oxidation in diabetic pericytes, consistent with some previous reports (8, 9). Since there is a lack of strategies to target pericytes for a translatable therapy, we generated AV-NG2p-OXR1. OXR1 is a well-known inhibitor of oxidative stress, and its effect on oxidation is through many downstream factors, including ROS (10), apoptosis-associated proteins (10), p53 signaling (11), p21 signaling (12), and histone arginine methylation (13). Therefore, overexpression of OXR1 in diabetic pericytes could have their antioxidant effects through all these pathways, which exactly targeted the impairment of diabetic pericytes in proliferation, control of apoptosis, and oxidative levels found in patients.

Our *in vitro* experiments using co-culture of genetically modified pericytes and HUVECs did not show a significant effect on the growth and tube formation of HUVECs by altering OXR1 levels in pericytes, but a significant effect on the migratory and branching potential of HUVECs, consistent with the role of pericytes in the maintenance of the vascular stability rather than its outgrowth. The restoration of the vascular stability in the diabetic wound by OXR1 expression in pericytes could facilitate wound healing through improved nutrient delivery and usage by the regenerating tissue in the wound. Moreover, it is possible that the OXR1 expression in pericytes may alter their interaction with inflammatory cells, especially macrophages, to further their effects on wound healing in a diabetic status. Indeed, a recent study showed that macrophages in a diabetic wound lost expression of an angiogenic factor, placental growth factor (PlGF), while re-expression of PlGF in diabetic macrophages significantly improved wound healing (14). Interestingly, pericytes are known to be regulated by PlGF and the vascular endothelial growth factor signaling pathway (15–17). Therefore, part of the effects of OXR1 expression in pericytes on diabetic wound healing may be through macrophages, and this question should be addressed in a future study.

Our *in vitro* results on the interaction between pericytes and ECs were further confirmed by the *in vivo* study in diabetic mice. In DB/DB mice at analysis, fasting blood glucose increased with age, and the beta cell mass also increased. Thus, the increase in beta cell number failed to compensate for the increased insulin resistance and beta cell dysfunction. The OXR1 expression in pericytes did not alter vessel density but improved blood flow to promote wound healing. Our work should provide a promising therapeutic strategy for diabetic wound healing through targeting pericytes, which deserves further investigation.

## Data availability statement

The original contributions presented in the study are included in the article/supplementary material. Further inquiries can be directed to the corresponding author.

## Ethics statement

The studies involving human participants were reviewed and approved by Wenzhou Medical University. The patients/participants provided their written informed consent to participate in this study. The animal study was reviewed and approved by Wenzhou Medical University.

## Author contributions

WJ, XC, LK, and CH are responsible for data acquisition and analysis; CH performed bioinformatics analysis. WJ, XC, LK, and CH are responsible for study conception and design, data acquisition, and analysis; CH wrote the manuscript; and all authors have read the manuscript and agreed with the publication. CH is responsible for funding and is the guarantor of the study. All authors contributed to the article and approved the submitted version.

## Funding

This work was supported by the Natural Science Foundation of Zhejiang Province (LWY20H020001).

## Conflict of interest

The authors declare that the research was conducted in the absence of any commercial or financial relationships that could be construed as a potential conflict of interest.

## Publisher’s note

All claims expressed in this article are solely those of the authors and do not necessarily represent those of their affiliated organizations, or those of the publisher, the editors and the reviewers. Any product that may be evaluated in this article, or claim that may be made by its manufacturer, is not guaranteed or endorsed by the publisher.
